# Questionnaire-based Prevalence of Food Insecurity in Iran: A Review Article

**Published:** 2017-11

**Authors:** Milad DANESHI-MASKOONI, Sakineh SHAB-BIDAR, Mahtab BADRI-FARIMAN, Erfan AUBI, Younes MOHAMMADI, Sadegh JAFARNEJAD, Kurosh DJAFARIAN

**Affiliations:** 1. Dept. of Community Nutrition, School of Nutritional Sciences and Dietetics, Tehran University of Medical Sciences, Tehran, Iran; 2. Dept. of Biostatistics and Epidemiology, School of Public Health, Tehran University of Medical Sciences, Tehran, Iran; 3. Social Determinants of Health Research Center, Hamadan University of Medical Sciences, Hamadan, Iran; 4. Dept. of Clinical Nutrition, School of Nutritional Sciences and Dietetics, Tehran University of Medical Sciences, Tehran, Iran

**Keywords:** Prevalence, Food insecurity, Questionnaire, Systematic review, Iran

## Abstract

**Background:**

Data on the questionnaire-based prevalence of food insecurity are needed to develop food and nutrition security studies and policies. The present study aimed to assess the questionnaire-based prevalence of food insecurity in Iran.

**Methods:**

A systematic search of cross-sectional studies were conducted on databases including PubMed, Google Scholar, Scopus, Magiran, Iranmedex, SID and Medlib up to 29 Oct 2015. Estimation of food insecurity prevalence was according to the instruments including 9-items-HFIAS, 18 and 6-items USDA (US-HFSSM) and Radimer/Cernel food security questionnaires. Pooled effect was estimated using random-effect model and heterogeneity was assessed by Cochran’s Q and *I*2 tests.

**Results:**

Thirteen articles included in the study based on screening and assessment of eligibility. The questionnaire-based prevalence of food insecurity was 49.2% (CI95%: 43.8–54.6). The according to sub-groups analysis, the food insecurity without and with hunger was 29.6% (CI95%: 25.7–33.6) and 19.2% (CI95%: 16–22.3), respectively.

**Conclusion:**

The about half of the population were food insecure. The food insecurity without hunger was more than the food insecurity with hunger. An ongoing food insecurity assessment system is needed to support evidence-informed policy and to plan interventions to increase the food security in different areas.

## Introduction

Food security as an state, related to the continuing availability of food around history, was defined as “availability at all times of adequate world food supplies of basic foodstuffs to sustain a steady expansion of food consumption and to offset fluctuations in production and prices” at the 1974 World Food Conference (1). The food security can be developed universally and for worldwide disastrous hazards that affect a significant diminution or deletion of customary agriculture, substitutive food changing is necessary (2).

At 1996 World Food Summit, the food security of individuals was accented rather than the nation. The *United States Department of Agriculture* (*USDA*) and the *Food and Agriculture Organization (FAO)* imply that food security occurs when all individuals, at all times, have physical and financial access to enough, safe and nutritive food to visit their nutritional needs and food priorities for an energetic and healthful life (3–5). The hunger or horror of starvation does not exist in the food secure peoples (6). In the other words, status of “finite or unsure availability of nutritionally sufficient and safe foods or restricted or uncertain ability to obtain favorable foods in socially admissible ways” is food insecurity (7).

The food security integrates a measure of versatility to future interruption or unavailability of basic foods because of different hazards including drought, shipping problems, fuel dearth, economic inconsistency, and wars.

In the years 2011–2013, about 842 million peoples were suffering from chronic hunger. The FAO reports almost 870 million peoples (12.5% of the worldwide population, or 1 in 8 people) were constantly malnourished in the years 2010–2012, mostly in developing countries (∼15%). However, rates of malnutrition in Asia and Latin America attained abatements that place them on way for reaching the Millennium Development Goal of bisecting the prevalence of malnutrition (8). The United Nations (UN), attended that consumption of vitamins and minerals is not enough (in about 2 billion peoples) (9). The rates of the hungry have been increased in nearly 30 million Indian since the mid-1990s (10). The according to reports of the USDA Economic Research Service (ERS), about half of the food insecurity is in Asia and likely near to 20% of these countries will be faced with hunger (11).

Food insecurity as chronic, seasonal and transitory forms exist at the household, region and nation levels and includes quantitative, qualitative, sociocultural and psychological dimensions (12). It results in insufficient dietary intakes, mental, psychological and behavioral problems in children and adults and low disease resistance (13, 14).

Today, the political security of countries is related to their food security and assessment of food insecurity prevalence and perspectives is necessary for attention to damaging outcomes (15–18).

The population numbers and characteristics are basis of all the planning and policymaking. The population is an important component of the socioeconomic status of any community and is influenced by socioeconomic policies. In recent decades in Iran, population growth has been declined because of different factors.

In attention to inconsistent data on the questionnaire-based prevalence of food insecurity at the national level and different survey methods in the most of Iranian studies (community-based) (19–23), this study designed to investigate the questionnaire-based prevalence of food insecurity in Iran as systematic review and meta-analysis.

## Materials and Methods

In order to find articles related to food insecurity prevalence, all domestic scientific database including Magiran, Iranmedex, SID, and Medlib as well as international databases including PubMed/Medline, ISI, Web of Knowledge, google scholar and Scopus were searched for published data related to food insecurity in Iran.

Search strategy was ((“food insecurity” OR “food security” OR “food supply” OR hungry OR starvation) AND (Iran OR Islamic Republic of Iran OR I.R.Iran)). Then in the local databases, “food insecurity (in Persian)” and “food security (in Persian)” were searched. Search results in different databases until 29 Oct 2015 were considered. In studies of hunger are checked food intakes and therefore term “hungry (in Persian)” was not search in local databases.

In the databases of PubMed, Google Scholar and Scopus, and the local databases of Magiran, SID, Iranmedex, and Medlib were found two hundred thirteen, six hundred, five, four hundred sixty-two, seventy, seventy-five and fifteen articles, respectively.

Checklist for data extraction included: English/Persian languages, cross-sectional studies, the sample size ≥100, an Iranian nationality, population representative sample (all groups of society, not a particular group), random sampling method, age ≥18 (or under 18 that the household food insecurity had been assessed), similar perception-based assessment instruments (including 6/18-items the United States Department of Agriculture [USDA] Household Food Security Survey Module [HFSSM], 9-items Household Food Insecurity Access Scale [HFIAS], and Radimer-Cernel food security questionnaires), and the number of excluded people from the study (or lack of cooperation by the end of the study) ≤10%.

Two researchers (MDM and MBF) independently screened the titles of all recorded citations, removing duplicate records and distinguishing potentially relevant studies for inclusion. Abstracts from selected citations were then independently reviewed by two researchers for further relevance, with full-text manuscripts retrieved as appropriate. In the disagreement cases, a third consultant acted as an intervener.

The extracted data were authors/year, location/setting, sample size and the food insecurity prevalence (total and without/with hunger). Meta-analysis was performed using the random effect model of Mantel-Haenszel, with available data presented in a forest plot. Standard error for each study was calculated using the binomial distribution formula. The presence of heterogeneity was determined by the chi2 test with a significance level of <0.1 combined with an *I*2 statistic for estimates of inconsistency within the meta-analysis. The *I*2 statistic estimates the percent of observed between-study variability due to heterogeneity rather than to chance and ranges from 0 to 100%. For this review, we determined that *I*2 values above 75 percent were indicative of significant heterogeneity warranting analysis with a random effect model as opposed to the fixed effect model to adjust for the observed variability between studies. Then the heterogeneity was further explored through subgroup analysis. Egger test was conducted to examine potential publication bias. Statistical software was STATA_11_.

## Results

**Fig. 1:**
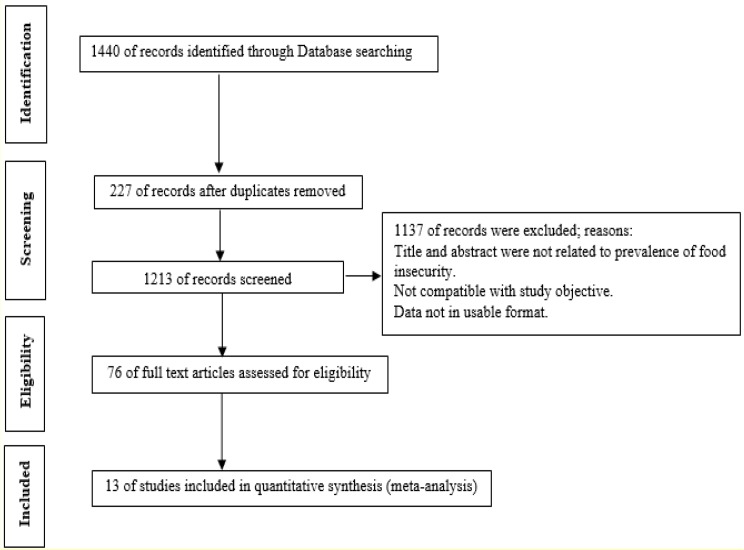
Flow diagram of the systematic literature search

Our systematic search found 1440 papers. Out of the number, 227 papers were duplicated. Moreover, we dropped out 1137 papers, because we found they were irrelevant based on screening of title and abstract. In addition, after reading the full text of the remained papers, we excluded 63 papers because of not having eligibility criteria. Finally, we reached to thirteen articles were eligible for meta-analysis phase. These thirteen articles include 15184 participants from seven different provinces of Iran (Fig. 1). The lowest prevalence of food insecurity was in Isfahan Province with 36.6% and the highest prevalence was reported in Tehran with 79%.

**Fig. 2:**
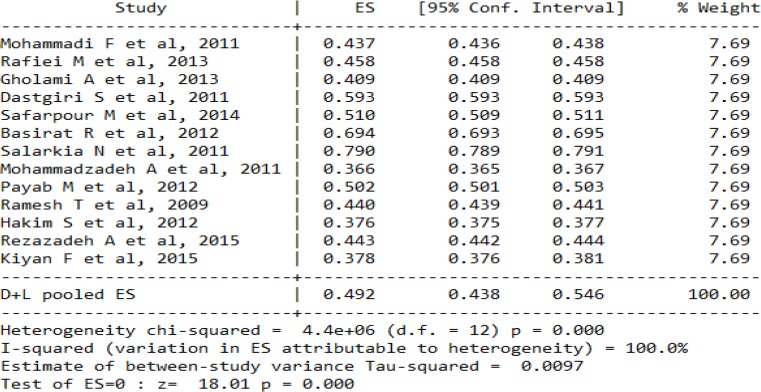

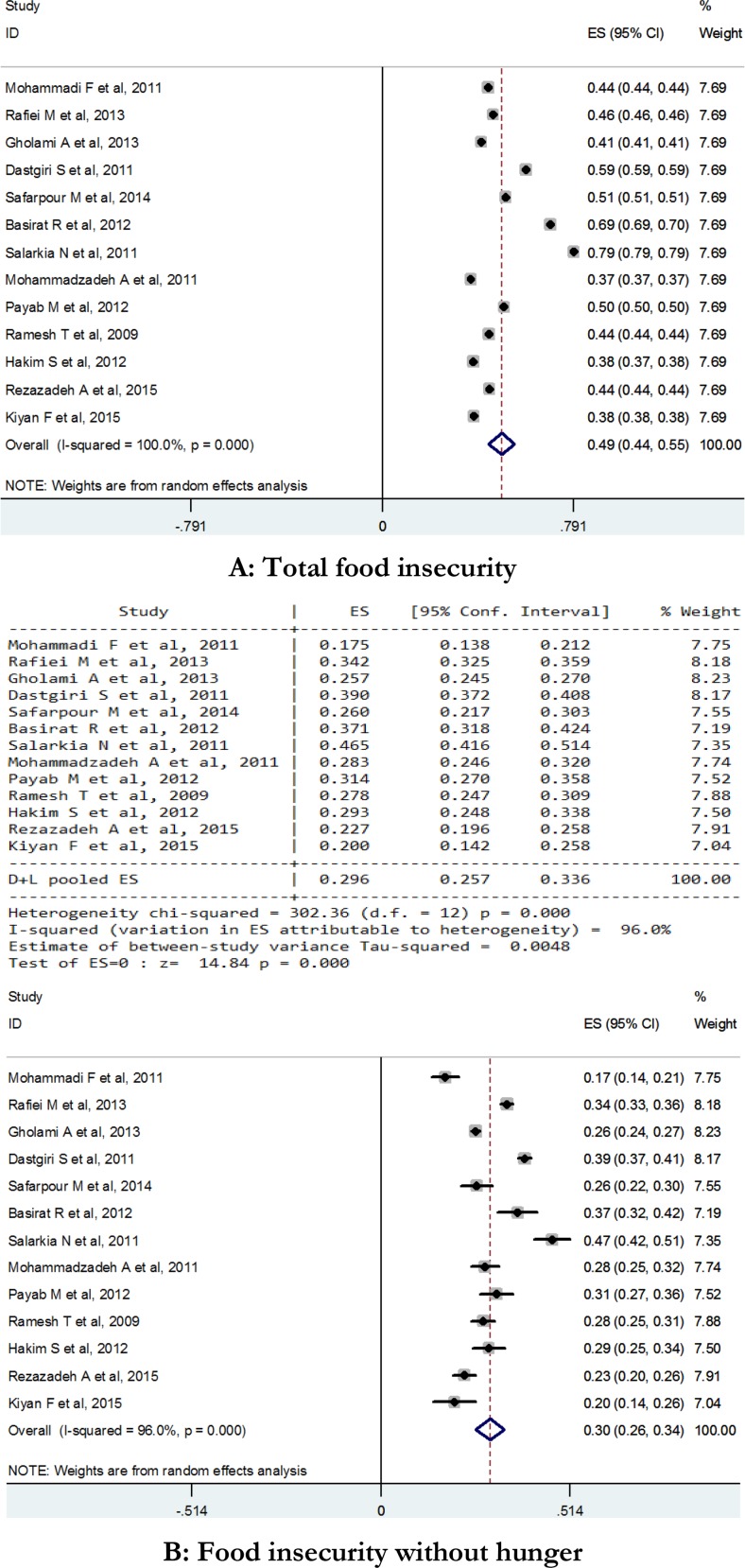

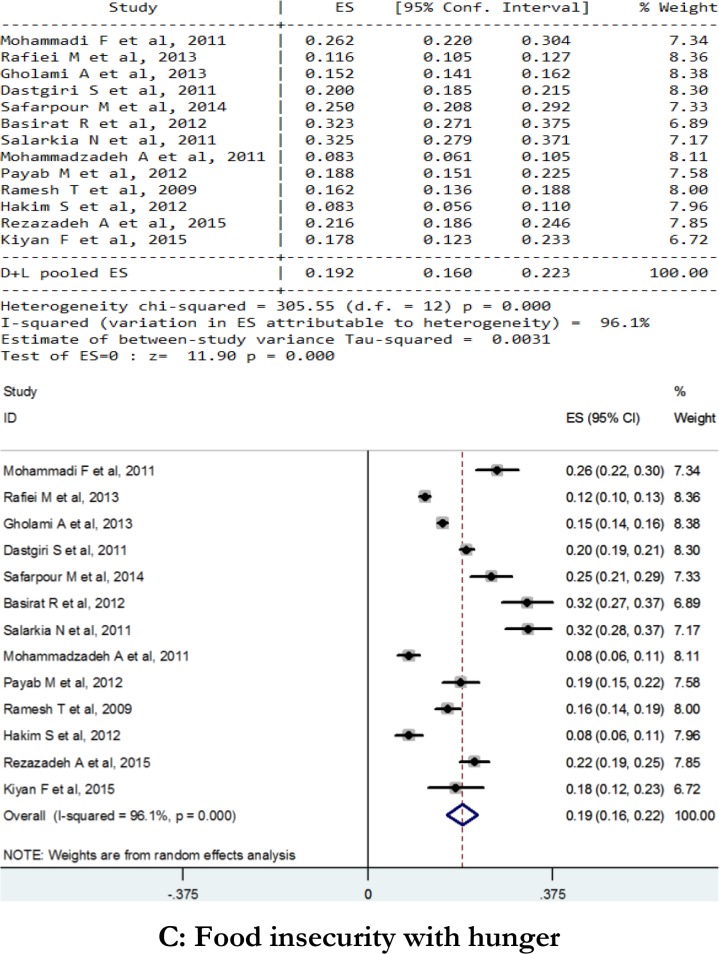
Forest plot of random effect model of mata-analysis for prevalence of food insecurity in Iran. Squares represented effect estimate of studies with their 95% confidence intervals with size of squares proportional to the weight assigned to the study in the meta-analysis. The diamond represents the overall results and 95% confidence interval of the random effect of the meta-analysis. **A**: Total food insecurity, **B**: Food insecurity without hunger, **C**: Food insecurity with hunger

**Fig. 3:**
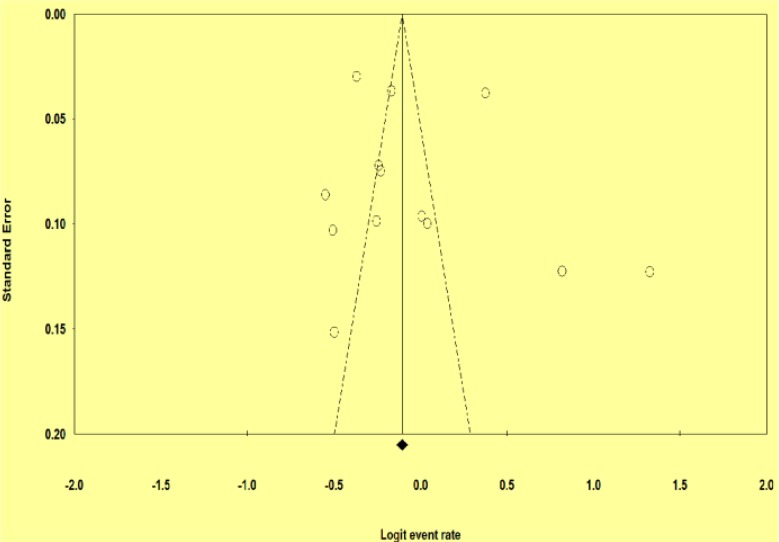
Funnel plot, using data from the 13 included studies in meta-analysis, with prevalence logit of food insecurity displayed on the horizontal axis and standard error of estimate on the vertical axis; symmetrical plot shows the presence of publication bias

Three studies were conducted in Tehran Province, and two studies in Isfahan and Fars Provinces. Provinces Mazandaran, east Azerbaijan, Chaharmahal and Bakhtiari, Alborz, Khuzestan, and Urmia had one studies in our meta-analysis. On the other hand, the newest and oldest studies were conducted in 2015 and 2009. The largest and smallest sample size belonged to Gholami’s study with 4647 participants and Kiyan’s study with 185 participants (Table 1). However, heterogeneity test was statistically significant, indicating the lack of homogeneity. Therefore, we used random effect model to deal with this issue. Pooled prevalence of food insecurity was 49.2% (CI95%: 43.8–54.6). In addition, our subgroup analysis by hunger status indicated that the prevalence of food insecurity without hunger was 29.6% (CI95%: 25.7–33.6) and with hunger 19.2% (CI95%: 16–22.3) (Fig. 2). The exploration of publication bias by funnel plot indicated asymmetry in the studies included in the meta-analysis (Fig. 3). Moreover, statistical test of publication bias by Begg’s test and Egger’s test did not reveal any significant evidence of publication bias (Begg’s test, *P*=0.62; Egger’s test, *P*=0.77).

**Table 1:** Delineation of the studies included in the meta-analysis of the questionnaires-based prevalence of food insecurity in Iran

***Studies***	***Setting***	***Samples (n)***	***Instrument***	***%FI (without & with hunger)***
Mohammadi F et al, 2011 (23)	Tehran (6 districts)	416	HFIAS	43.7 (17.5, 26.2)
Rafiei M et al, 2013 (22)	Isfahan	3000	18-USDA	45.8 (34.2, 11.6)
Gholami A et al, 2013 (21)	Neyshabour	4647	6-USDA	40.9 (25.74, 15.15)
Dastgiri S et al, 2011 (20)	Northwest	2911	6-USDA	59.3 (39, 20))
Safarpour M et al, 2014 (40)	Bandar-Anzali	400	18-USDA	51(26, 25)
Basirat R et al, 2012 (41)	Farokhshahr	314	Radimer	69.4 (37.1, 32.3)
Salarkia N et al, 2011 (42)	Varamin	400	HFIAS	79 (46.5, 32.5)
Mohammadzadeh A et al, 2011 (43)	Isfahan	580	18-USDA	36.6 (28.3, 8.3)
Payab M et al, 2012 (44)	Rey (Tehran)	430	18-USDA	50.2 (31.4, 18.8)
Ramesh T et al, 2009 (45)	Shiraz	778	18-USDA	44 (27.8, 16.2)
Hakim S et al, 2012 (46)	Dezful	400	18-USDA	37.6 (29.3, 8.3)
Rezazadeh A et al, 2015 (47)	Urmia	723	HFIAS	44.3 (22.7, 21.6)
Kiyan F et al, 2015 (48)	Alborz	185	HFIAS	37.83 (20, 17.83)

## Discussion

This study demonstrated heterogeneity for the prevalence estimates of food insecurity. As shown in funnel plot, an asymmetrical plot is occurred due to low methodological quality of smaller studies, so results are biased towards high quality studies with larger sample size (24).

After random effect model meta-analysis, the questionnaire-based prevalence of food insecurity in the Iran was 49.2% (without hunger 29.6% and with hunger 19.2%). In other words, less than half of the households are facing with food insecurity. In previous study, household income/expenditure-based prevalence of moderate to severe food insecurity was reported about 10% and experiential/perception-based prevalence of mild, moderate and severe food insecurity were 28.6%, 14.9% and 6.0%, respectively (25). The current nutritional transition of Iran has changed nutritional status within the households significantly. On the other hand, diets with low nutrient density at different economic levels, overeating and obesity are evident among more than a third of the population, while food insecurity is also evident as a proportion of the Iranian (26).

The questionnaire-based prevalence of food insecurity without and with hunger was 29.6% and 19.2%, respectively, in the years 2009 to 2015. These rates were higher than the initial reports (8.9% and 10% respectively in 1994 and 10.5% and 13% respectively in 2004) (27, 28) and the previous meta-analysis of complete national food consumption surveys in 1993 and 2004 (8.5% and 9.2% respectively in 1994 and 9.3% and 9.3% respectively in 2004) (25). The high accuracy of obtained results from our meta-analysis is due to the application of weighting methods and a heterogeneity test for appraising the whole prevalence.

The reasons of food insecurity were not checked in our study. Yet, season and food access in urban communities are independent of each other. The economic factors influence food access, food cost inconstancy, and are the main agents affecting food insecurity in Iran (29).

In attention to a very small number of perception-based studies in the Eastern Mediterranean area, comparing our results and other countries of area is not feasible. However, the food insecurity ranking of Iran is in the middle according to comparing the food insecurity prevalence in Iran with other data (“based on food production per capita, the ratio of total export earnings and food imports, calories per capita and protein per capita, and nonagricultural population share”) whereas Iraq, Yemen and Sudan were at the highest levels of penury and food insecurity (30).

The food insecurity prevalence without and with hunger from the previous meta-analysis of the perception-based studies were 28.5% and 20.9%, respectively, while in our study, these rates were slightly higher (29.6%) and lower (19.2%), respectively. The rates of the comprehensive national food consumption surveys argued above are much lower than these values. Likely, this difference is related to the character of perception-based questionnaires that assess bothers about food and attitudes, while recall-based surveys assess energy intake inadequacy because of food insecurity. The participant’s overestimation in the perception-based surveys is possible that should be taken into consider. Inclusion criteria of our study were more detailed and different. Accordingly, number and type of selected articles for this analysis were lower and more accurate of previous meta-analysis.

The results of perception-based surveys in developmentally similar countries, for example Brazil, showed that the food insecurity prevalence without and with hunger were 23.1% and 14.4%, respectively (31). However, our rates are lower than penurious developing countries, such as Malawi, that the food insecurity prevalence with hunger was 48.1% (32). In Jordan, food insecurity data of national surveys on food availability and energy intake are similar to Iran (30), but perception-based rates (in northern Jordan) were more than Iran (32.4% food insecurity prevalence with hunger) (33). The perception-based prevalence of food insecurity in developed countries is very lower, for example in Canada and the United States (US), where that food insecurity values with hunger were 14.6% and 7.8%, respectively (34, 35).

The US-HFSSM or HFIAS methods were used mainly to assess the food insecurity in our study and the other perception-based surveys. The fundamental approach for both measures is the identical. Nevertheless, the expected aim and range of usage for each instrument is dissimilar. The HFSSM was expanded solely for use in the US, whereas the HFIAS was expanded to give an equal cross-cultural assessment of food insecurity in funds-lean regions within developing countries. The pertinence of these tools for use in the Iran should check accurately. Even so, according to other studies, the choice of tool for a specific population is likely important (36).

The WHO expresses that three columns explain food security: availability, access and use of food (37). The FAO attaches a fourth column: the stability of the first three parameters of food security throughout of time (8). The World Food Security Summit in 2009 declared that the “four dimensions of food security are availability, access, use, and stability” (38). Stability parameter can change over time and will influence other dimensions. Likely, the potential reasons of food insecurity in Iran are related to stability.

Our study had a few limitations. The data of our systematic review and meta-analysis were extracted from only perception-based studies (questionnaire-based). The included studies were mainly related to the areas of the center to the north and some provinces of the Iran. The separate data of food insecurity in different provinces, climates and ethnic groups and rural areas were not available. Accordingly, it was not possible to assess food insecurity prevalence in urban and rural areas separately.

Our study is the first systematic review and meta-analysis of researchers on the questionnaire-based prevalence of food insecurity in the Iran. In attention to the lack of an ongoing food security assessment system in the Iran, such assessment methods are needed to support evidence-informed policy and to design interventions to increase food security in various areas of the country.

National food consumption has been surveyed every 10 yr in the Iran and has given valorous data for policymaking. A valid perception-based (or questionnaire-based) strategy be used over time at the national level to give analogous data in shorter interims because to the cost and time-consuming character of food consumption studies (25). However, the recent studies of food security in Iran are mostly related to the health status. The continuing project of Food Insecurity and Vulnerability Information and Mapping Systems (*FIVIMS*) in Iran has been implementing to give the fundamental data and geographical maps of food insecurity and vulnerability (39).

## Conclusion

According to questionnaire-based (or conception-based) method, about half of Iranian peoples were food insecure. However, food insecurity without hunger was more of than food insecurity with hunger. The food insecurity prevalence in the Iran has been rising and due to different consequences for the individual, society, and government, it should be seriously considered and planners take steps to increase food security. The further studies are needed to assess status of food insecurity in Iran using valid and reliable instruments.

## Ethical considerations

Ethical issues (Including plagiarism, informed consent, misconduct, data fabrication and/or falsification, double publication and/or submission, redundancy, etc.) have been completely observed by the authors.
